# Impact of a School-Based Multicomponent Positive Psychology Intervention on Adolescents’ Time Attitudes: A Latent Transition Analysis

**DOI:** 10.1007/s10964-021-01562-5

**Published:** 2021-12-31

**Authors:** Claudia Tejada-Gallardo, Ana Blasco-Belled, Carles Alsinet

**Affiliations:** 1 Universitat de Lleida, Avinguda de l’estudi general, 4, 25001 Lleida, Spain; 2 Universitat de Girona, Pujada de Sant Domènec, 9, 17004 Girona, Spain

**Keywords:** Adolescents, Time attitudes, Profiles, Positive psychology, Multicomponent positive interventions, School

## Abstract

Time attitudes, which refer to positive and negative feelings towards the past, present, and future, are a salient phenomenon in the developmental stage of adolescence and have been related to better well-being. Positive feelings towards time can be promoted in the school setting through empirically validated positive psychology interventions. However, the extent to which these interventions impact the time attitudes of adolescents remains unknown. The current study investigated the influence of a multicomponent positive psychology intervention on adolescents’ transitions between time attitude profiles and how these transitions are related to their emotional, social, and psychological well-being. Participants consisted of 220 (*M* = 14.98; 47.3% female) adolescents from two Spanish high schools who participated in the six-week Get to Know Me+ program. Adolescents’ time attitudes and well-being were measured via the Adolescents and Adult Time Inventory–Time Attitudes and the Mental Health Continuum–Short Form, respectively, at pre- and postintervention. Participants were clustered in different profiles through a latent profile analysis, and the transitions were analyzed using a latent transition analysis. Five profiles were identified (*negative*, *present/future negative*, *past negative*, *optimistic*, and *positive*), and results indicated that adolescents who participated in the intervention were more likely to transition to positive profiles (*optimistic* and *positive*) and generally reported higher well-being, especially those in the *negative*, *present/future negative*, and *optimistic* profiles. Preliminary evidence showed that school-based multicomponent positive psychology interventions can have a positive impact on adolescents’ feelings towards time and well-being.

## Introduction

Over the last two decades and with the emergence of positive psychology, the study of mental health has gained attention, as reflected by an exponential growth in research and an increased focus on this topic in practice. The traditional focus on negative aspects and the treatment of mental illness has evolved towards a more comprehensive understanding of mental health. The latter is conceived as the presence of well-being and the absence of psychopathology (Keyes, 2009). Adolescence can entail the onset of many mental adult disorders (Kessler et al., 2005), suggesting that the promotion of well-being at early stages may prevent future adult psychiatric disorders. In the educational context, positive education emphasizes the promotion of positive emotions, relations, and character strengths to foster the happiness and well-being of children and adolescents for educational purposes (Bernard & Walton, 2011; Seligman et al., 2009). According to this, programs that focus on the abovementioned aspects should be well-received by educational institutions. Nevertheless, there are still many obstacles in introducing these programs as part of school curricula (Weissberg et al., 2013). Adolescence is also a stage in one’s biological and psychological developmental (Burger & Samuel, 2017) wherein time attitudes, which refer to positive and negative feelings towards time (past, present, and future), are a salient phenomenon. Previous research advocated for an association between time attitudes and emotional, social, and psychological well-being (Tejada-Gallardo et al., 2021). Studies investigating the effects of positive psychology education programs have shown positive results on well-being (e.g., Shoshani et al., 2016). In fact, time attitudes are likely to be responsive to positive psychology interventions because positive feelings towards time are predicted by beneficial variables at school, such as positive teacher-student relationships and satisfaction at school (Froiland et al., 2019). With the aim to broad the research field of adolescents’ time attitudes and well-being, this study attempted to evaluate the impact that a school-based multicomponent positive psychology intervention had on adolescents’ positive and negative feelings towards time and whether this intervention could influence their emotional, social, and psychological well-being.

### Time Attitude Profiles and Well-Being in Adolescents

Mello and Worrell (2015) presented a conceptual model of time perspective for adolescents. This model proposes time perspective as a cognitive and motivational construct that originates in the thoughts of individuals and leads to decision-making processes and engagement in specific behaviors. Time perspective is also conceptualized as a multidimensional construct that encompasses five dimensions (i.e., time attitudes, time orientation, time relation, time frequency, and time meaning) in the three time frames (i.e., past, present, and future). The time attitude dimension is the most commonly studied component of time perspective; it refers to individuals’ positive and negative feelings towards the past, present, and future (Mello & Worrell, 2015). Previous research on adolescents has employed time attitudes to investigate several developmental outcomes through a person-centered approach (e.g., Konowalczyk et al., 2019), which involves identifying and grouping individuals who share particular attributes. Latent profile analysis is a latent variable approach that focuses on identifying latent subpopulations (i.e., profiles) from a population based on a set of continuous variables (i.e., indicators; Wang & Hanges, 2011). In the present study, the latent profile analysis is used to identify individuals who display similar positive and negative feelings towards the past, present, and future, and the results of this analysis can therefore be used to describe the distribution of time attitudes across individuals (Lanza et al., 2010).

Adolescence is an important period in the emergence of mental illnesses, especially with regard to the onset of anxiety and depression symptoms (Paus et al., 2008). It is not only the presence of mental disorders at this developmental stage that is alarming, as the instability of their well-being, which tends to decrease from early to late adolescence (González-Carrasco et al., 2017). Adolescents’ positive profiles have been related to beneficial outcomes, such as higher well-being and lower psychological distress (Tejada-Gallardo et al., 2021), suggesting that positive feelings towards the three time frames lead to optimal psychological functioning. Several profiles have emerged throughout the literature on time attitude profiles, for instance, negatives, present/future negatives, past negatives, moderately-negatives, ambivalents, optimists, balanced, and positives (Andretta et al., 2014; Tejada-Gallardo et al., 2021; Worrell et al., 2019). However, it seems that adolescents’ time attitude profiles neither follow a common pattern of clusters (Tejada-Gallardo et al., 2021) nor are stable over time, especially when these profiles are related to developmental outcomes characteristic of this particular stage (McKay et al., 2018). For instance, some scholars observed a high frequency of transitioning (91.2%) among profiles between ages 13 and 15 (Konowalczyk et al., 2018). Other authors also reported a high percentage of adolescents who transitioned from one profile to another over time, with the change from a positive to a negative profile being the most common (Wells et al., 2018). In short, the development of time attitudes, which emerge in adolescence, is meaningfully related to mental health outcomes. Hence, the maintenance of or transition to positive profiles is somehow beneficial for this specific period of life (McKay et al., 2018). Given that this is the case, interventions aimed at improving adolescent’s well-being by focusing on their positive feelings towards the past, present, and future may have an impact in terms of influencing transitions of individuals to more positive profiles.

### Positive Psychology Interventions in the School Setting

Approximately 3 million people in Spain are diagnosed with depression every year, and this condition typically has its onset at the age of 15 (Spanish Ministry of Health, Consumer Affairs, and Social Welfare, 2017). This age is of particular importance given that in the Spanish educational system, it is considered a key transitional stage where adolescents should decide whether they wish to continue studying or join the professional world. The promotion of well-being in adolescents can be an effective strategy to ensure young adult transitions and future adult positive mental health (O’Connor et al., 2017). Even though the prevention of mental illnesses is central to psychologists’ work, building and promoting well-being should be of equal importance, as adolescents may not have optimal functioning regardless of the absence of any mental disorder (Suldo et al., 2014). Promoting well-being may thus help young people to meet the significant demands they face as they move into young adulthood (Schulenberg et al., 2004). Considering this issue in the context of the school setting, there is growing interest in the potential to promote healthy pathways into adulthood through school-based interventions targeting well-being, an approach that is often referred to as positive education (O’Connor et al., 2017). Positive education emerged alongside the positive psychology movement, which involves the application of empirically validated interventions and programs that have a positive impact on well-being. Positive interventions have also been introduced in the school setting with the aim of allowing children and adolescents to thrive both psychologically and academically (Green et al., 2011). In short, positive education can be understood as the application of positive psychology interventions in educational settings, and it is considered an approach that promotes well-being and academic learning (Seligman et al., 2009).

The school institution is considered a vital place of growth that affects life at home, the community, and future workplace (Seligman et al., 2009). Schools are excellent settings for delivering positive psychology initiatives because adolescents spend a great part of their waking time at school but also because these programs can be freely accessed by all adolescents, not only those that have the wealth and time to access them via private practices. However, reform at the policy level will be required to overcome the disconnection between the growing evidence as to the efficacy of school-based positive interventions and the relevant obstacles (White, 2016). One type of these interventions, multicomponent positive psychology interventions, are based on a variety of individual exercises targeting two or more theoretically relevant well-being components that resemble the key elements of positive education and are conducted within an integral program. When compared to single-component positive psychology interventions, multicomponent positive psychology interventions have been found to have more pronounced and long-term effects (Rusk et al., 2018; Seligman, 2011, 2018).

### A School-Based Multicomponent Positive Psychology Intervention: The Get to Know Me+ Program

Get to Know Me+ is a multicomponent positive psychology school program consisting of three modules targeting different components of well-being across the three time periods: (1) focusing on the positive emotions of the present, (2) turning back to the positive emotions of the past, and (3) moving forward to the positive emotions of the future. An individual’s behavior is not determined solely by their present situation, as one’s mood is affected by personal hopes, goals, and views of the past (Mello & Worrell, 2015). Subjective well-being represents people’s evaluations of their lives, including cognitive evaluations (i.e., life satisfaction) and affective evaluations (i.e., positive and negative emotions; Tomlinson et al., 2017). These components focus not only on present life experiences but also on past recollections and future expectations regarding one’s life (Cunningham et al., 2014). Hence, the positive or negative feelings that adolescents have towards time also influence their emotional state and well-being (Tejada-Gallardo et al., 2021). The Get to Know Me+ program incorporates several aspects that emphasize the promotion of adolescents’ well-being through the three time frames. For instance, gratitude practices may boost positive feelings towards the past (Seligman et al., 2005), signature strengths practices may develop positive feelings towards the present (Proctor et al., 2011), and reflecting on one’s best future self may promote positive feelings towards the future (Sheldon & Lyubomirsky, 2006). Each session consisted of three parts with an introductory flow activity, a central activity to put in practice the principle of well-being, and the closing of the session. A more detailed description and plan of the program can be found in the Appendix.

## The Current Study

Longitudinal studies have revealed that time attitude profile membership is susceptible to change over time, especially in adolescents, and cross-sectional studies have suggested that a positive time attitude profile is optimal in terms of well-being. Given the evidence and the lack of research on the impact of multicomponent positive psychology interventions on adolescents’ time attitude profiles, the goal of the present study was to investigate to which extent a multicomponent positive psychology intervention (Get to Know Me+) can influence the probability of adolescents’ transitions towards more adaptive time attitude profiles and the impact of these transitions on well-being. Although multicomponent positive psychology interventions are an effective strategy by which to increase well-being in the school setting, whether they can prompt transitions towards more positive profiles and the possible benefits of the transitions in terms of the emotional, social, and psychological well-being of adolescents remains unknown. Hence, this study presents an exploratory investigation of the topic. It has been hypothesized that the multicomponent positive psychology intervention will help adolescents to transition to more positive profiles (compared to the control group) and that the individuals with positive profiles in the intervention group will report higher emotional, social, and psychological well-being.

## Methods

### Participants and Procedure

Participants were 220 students^1^ in the 9^th^ grade (M_age_ = 14.98 years, *SD* = 0.62; 47.3% female) from two different high schools from west Catalonia (Lleida, Spain) that voluntarily participated in the program, which was conducted from October 2019 to December 2019. An invitation to participate in the study was given to different high-schools from the city of Lleida. Given that the intervention would imply re-scheduling some classes, only two high schools voluntarily accepted the invitation and were recruited to participate in the study. Further demographic characteristics of participants are presented in Table 1. Students needed to present an informed consent from their parents in order to participate in the research study. This informed consent stated that they could withdraw the study at any time. Of the total sample, 135 adolescents were randomly allocated to the control group (placebo) and 85 to the intervention group. All participants in the control group, with the exception of one, completed the baseline assessment and the posttest assessment; thus, a total of 134 participants were ultimately retained for the statistical analyses. Regarding the intervention group, the adolescents’ responses were only considered if they had attended at least four of the six sessions. Six participants dropped out; thus, 79 participants were ultimately retained for the statistical analyses (total retention rate of 96.81%). The attrition rate of the present study is considered to be very low as dropout rates of other school interventions tend to be higher (e.g., 30%; Chacko et al., 2016). The assessments and intervention were delivered by two psychologists and doctoral students who were specifically trained for this purpose. The adolescents completed a pretest assessment (one week prior to the beginning of the program) and posttest (one week after the end of the program). The control group participated in a one-day individual session about their character strengths which was also delivered by the psychologists and doctoral students. The present study was approved by the University Ethics Committee under the code CEIC-2157.

**Table 1:** Sample demographics reported at baseline assessment

	Intervention (*N* = 85)		Control (*N* = 135)	
Demographic	*N*	%	*N*	%
*Gender*				
Female	44	51,8	60	44,4
Male	41	48,2	73	54,1
Other	0	0	2	1,5
*Ethnicity*				
Hispanic, Latino or other	6	7,0	8	5,9
Spanish origin	69	81,2	118	87,4
Not Hispanic	10	11,8	9	6,7
*Socioeconomic status*				
Low	16	18,8	36	26,7
Average	56	65,9	86	63,7
High	13	15,3	13	9,6
*Family composition*				
Both parents together	59	69,4	111	82,2
Only one of the parents	20	23,5	24	17,8
Other family member	6	7,1	0	0

### Measures

#### Time attitudes

Time attitudes were measured with the Adolescent and Adult Time Inventory–Time Attitudes Scale (AATI–TA; Mello & Worrell, 2007; Spanish adaptation of Mello et al., 2010). The AATI–TA consists of six subscales that assess past positive, past negative, present positive, present negative, future positive, and future negative attitudes. The scale includes 24 items, which participants respond to using a 5-point Likert scale (1 = *totally disagree*, 5 = *totally agree*). The Cronbach’s *α* reliability estimates for the present study were 0.88 (past positive), 0.92 (past negative), 0.89 (present positive), 0.73 (present negative), 0.87 (future positive), and 0.75 (future negative) at Time 1 and 0.89 (past positive), 0.90 (past negative), 0.91 (present positive), 0.73 (present negative), 0.89 (future positive), and 0.84 (future negative) at Time 2. The following sample items are representative of each subscale: “I have very happy memories of my childhood” for past positive, “I wish that I did not have the past that I had” for past negative, “I am happy with my current life” for present positive, “I am not satisfied with my present” for present negative, “Thinking about my future excites me” for future positive, and “Thinking about my future makes me sad” for future negative.

#### Well-being

Well-being was assessed using the Mental Health Continuum–Short Form (MHC–SF; Keyes et al., 2008; Spanish adaptation of Echeverría et al., 2017). The MHC–SF assesses an individual’s emotional, social, and psychological well-being during the last month. This scale includes 14 items, which participants respond to using a 6-point Likert scale (1 = never, 6 = every day). The following sample items are representative of each subscale: In the past month, “how often did you feel happy?” for emotional well-being; “how often did you feel that you had something important to contribute to society?” for social well-being; and “how often did you feel that you liked most parts of your personality?” for psychological well-being. The Cronbach’s α reliability estimates for the MHC–SF were 0.77 (emotional well-being), 0.71 (social well-being), and 0.79 (psychological well-being) at Time 1 and 0.87 (emotional well-being), 0.81 (social well-being), and 0.82 (psychological well-being) at Time 2.

### Data Analytic Plan

#### Preliminary analysis

A series of preliminary analyses were performed (detailed information is provided in Supplementary Material 1). All analyses were conducted with the maximum robust likelihood estimator (MLR) in Mplus 7.2 (Muthén & Muthén, 2012). Preliminary measurement models were first estimated at both time points to identify the optimal model for further analyses and their longitudinal measurement invariance across time points (Millsap, 2011). Items of time attitudes (i.e., profiles) and well-being (i.e., outcomes) scales were estimated with a confirmatory factor analytic (CFA) model that included six first-order factors for time attitudes (past positive, past negative, present positive, present negative, future positive, and future negative) and three first-order factors for well-being (emotional, social, and psychological well-being). Detailed information concerning the models, measurement invariance, and correlations is presented in Supplementary Material 1.

Factor scores were extracted rather than using scale scores to estimate the profiles and their relations with the outcomes. The use of factor scores in the following analysis made it possible to partly control for measurement error (Skrondal & Laake, 2001). To ensure comparability across the two time points, factor scores from the most invariant longitudinal measurement model were extracted (Millsap, 2011).

#### Latent profile analysis and latent transition analysis

At both measurement time points and for each group (i.e., control and experimental), one to seven latent profiles solutions were estimated based on the six-time attitude factors as profile indicators. Means and variances for these indicators were free to vary across profiles (Diallo et al., 2016; Peugh & Fan, 2013). The section titled Supplementary Material 1 presents detailed information concerning the model comparison procedures used to select the optimal number of profiles.

Once the optimal number of profiles was identified at both measurement time points and for each group, the two-time multigroup specific models were combined into a longitudinal latent profile analysis to verify the extent to which these solutions were similar across time points. This strategy, which is a combination of the longitudinal profile similarity test suggested by Morin and Litalien (2017) and the test of profile similarity across multiple groups developed by Morin et al. (2016), was used to determine whether the same number of profiles could be identified across time points and groups (i.e., within-sample stability; Kam et al., 2016). First, it was examined whether the same number of profiles was identified across time points and groups (*configural* similarity). This model was free of constraints and served as a baseline model for comparison with subsequent models in which restraints were sequentially incorporated. Second, *structural* similarity was tested by including equal constraints across time points and groups on the means of the profile indicators. This step was used to verify whether the shapes of profiles were similar or stable over time and between groups. Third, *dispersion* similarity of the profiles was examined by including equality constraints on the variances of the profiles across time points and groups. This step was used to test whether the within-profile similarity remained stable over time and between groups. Fourth, *distributional* similarity of the profiles was tested by constraining equality class probabilities across time points and groups. This last test was used to determine whether the prevalence of profiles was stable over time and between groups. The hypothesis of profile similarity will be supported when the values for at least two indices of the consistent Akaïke information criterion (CAIC), the Bayesian information criterion (BIC), and the sample-size adjusted BIC (aBIC) are lower for the model containing more equality constraints (Morin et al., 2016).

The most similar model from the previous procedure was then retained and converted into a longitudinal latent transition analysis to estimate the within-person stability in profile membership across time points and groups (i.e., whether adolescents remained in the same profile over time and which profile transitions were observed for each group; Kam et al., 2016). To facilitate the labeling of profiles, time attitude factor means were standardized into T-scores (*M* = 50, *SD* = 10), and the criterion of ±0.5 *SD*s around the mean for each factor was followed, meaning that positive values were above 0.5 *SD* and negative values were below 0.5 *SD* (e.g., Worrell et al., 2019). This conversion yields comparable scores that are easily interpretable. Finally, outcomes were incorporated into the final latent transition analysis.

#### Outcomes of profile membership

The outcomes (emotional, social, and psychological well-being) were added to the final retained model. First, it was allowed the outcomes to freely differ across profiles and time points for each group. Second, explanatory similarity of the profiles was tested by constraining the within-profile means of the outcomes to be equal across time points. The model constraint function of Mplus was used to statistically test the mean differences between each pair of profiles based on the multivariate delta method (Kam et al., 2016; Raykov & Marcoulides, 2004). All the data and syntaxes necessary to replicate the results of the presented analyses are available to download in the open OSF repository.

## Results

### Profile Selection and Latent Transition Analysis

As a first step, the procedure used to determine the optimal number of profiles is detailed in the section titled Supplementary Material 1 and the results for the latent profile analysis models are presented in Supplementary Material 2 (Tables S3 and S4). The results suggested that the AATI–TA five-profile solution was the best option. The two-wave longitudinal latent profile analysis model of *configural* similarity was first estimated with a five-profile solution to verify the extent to which the same profiles were similar across measurement time points. The fit indices for all longitudinal models are presented in Table 2. Compared to the initial model of *configural* similarity, the next two models resulted in lower values for all fit indices, thereby supporting the *structural* and *dispersion* similarity of the five-profile solution over time. Finally, the model of *distributional* similarity resulted in higher values for all fit indices when compared to the previous model, which indicated the within-profile similarity of profiles over time. These results allowed to proceed with the following step, which consists of identifying and describing the profiles from the *dispersion* similarity model.

**Table 2:** Results from the longitudinal latent profile analyses and latent transition analyses estimated on the full sample

	LL	#*fp*	Scaling	AIC	CAIC	BIC	aBIC	Entropy
*Longitudinal Latent Profile Analyses*								
Configural similarity	−2071.475	153	1.389	4448.949	5116.227	4963.227	4478.415	0.946
Structural similarity	−2141.496	63	1.635	4409.496	4694.257	4631.257	4421.629	0.924
Dispersion similarity	−2155.023	45	1.704	4400.046	4596.304	4551.304	4408.712	0.922
Distributional similarity	−2158.030	41	1.816	4398.060	4696.873	4555.306	4410.719	0.922
Latent Transition Analysis (Dispersion)	−2044.741	69	1.364	4227.483	4528.412	4459.412	4240.771	0.945
*Explanatory Similarity*								
Free relations with outcomes	−2886.781	54	0.580	6451.561	7645.039	7591.039	6516.847	0.982
Equal relations with outcomes	−3157.527	33	1.116	6423.054	6637.564	6604.564	6433.454	0.952

*Note*. LL model LogLikelihood, #fp number of free parameters, Scaling scaling factor, AIC Akaïke information criteria, CAIC consistant AIC, BIC Bayesian information criteria, aBIC sample-size adjusted BIC

**Fig. 1:**
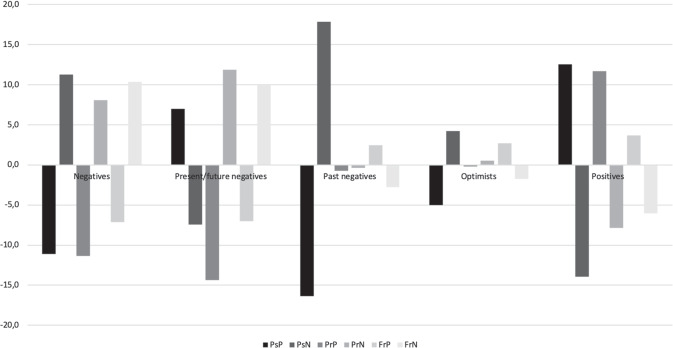
Final 5-profile solution identified in the study at both time-points and groups*. Note*. PsP past positive, PsN past negative, PrP present positive, PrN present negative, FrP future positive, FrN future negative

The model of *dispersion* similarity was then retained and converted into a complete latent transition analysis model. This final model, which resulted in high levels of classification accuracy (0.95), is illustrated in Fig. 1, and the exact within-profile means of the latent indicators are reported in the section titled Supplementary Material 2 (Table S5). After standardizing the means into T-scores, Profile 1 constituted 11.26% of the test sample (*n* = 24) and was labeled as (past, present, and future) *negative* due to the high mean scores on every negative subscale, with all standardized scores ranging between 0.5 and 1.5 *SD* and positive mean scores ranging between −0.5 and −1 *SD*. Profile 2 constituted 17.37% of the test sample (*n* = 37) and was labeled *present/future negative* due to the high mean scores on present and future negative subscales, which were between 1.0 *SD* and 1.5 *SD* above the mean. The mean scores for the present and future positives subscales were between −0.5 and −1.5 *SD*. Profile 3 constituted 9.85% of the test sample (*n* = 21) and was labeled *past negative* due to a high mean score on the past negative subscale, which was more than 1.5 *SD* above the mean, and its past positive mean score, which was more than 1.5 *SD* below the mean. The mean scores for the present and future subscales (positive and negative) were all close to the mean (<±0.5 *SD*). Profile 4 constituted 15.49% of the test sample (*n* = 33) and was labeled *optimistic* due to its negative past mean score of around 0.5 *SD*, present (positive and negative) mean scores close to the mean (<±0.5 *SD*), and a future positive mean score of around 0.5 *SD*. Profile 5 constituted 46% of the sample (*n* = 98) and was labeled *positive* due to high mean scores on the past and present positive subscales, which were more than 1 *SD* above the mean, and the future positive subscale, which was 0.4 *SD* above the mean. By contrast, the mean scores of the negative subscales were between 0.5 *SD* and 1.5 *SD* below the mean. All profiles retained the labels used in previous studies because the results yielded similar profiles with similar scores (Konowalzyk et al., 2019; Worrell et al., 2019).

The next step consisted in the analysis of the transitions between time attitudes profiles. The *dispersion* similarity model was converted into a latent transition analysis and showed a moderate level of within-profile stability in profile membership for both groups, which suggests that a considerable number of participants remained in the same profile at Time 2. More specifically, between 10–60% of adolescents in the control group and 15–65% of those in the experimental group changed profiles over time (Table 3). Regarding the control group, the *negatives* (71.1%), *present/future negativ*es (78.5%), and *positives* (87.1%) were the profiles that showed more stability over time, whereas, in the intervention group, the *negatives* (84.7%) and the *positives* (77%) were the most stable. The corresponding stability rates were lower in the intervention group for the *present/future negatives* (67.6%), *past negatives* (control: 65.2%; intervention: 36.3%), and *optimists* (control: 39.7%; intervention: 69%). The *past negative* profile (control: 12.5%; intervention: 5.7%) received fewer transitions from other profiles, followed by the *present/future negatives* (22.6%) in the intervention group, and the *optimists* (28.4%) in the control group. By contrast, the profiles to which individuals transitioned the most were the *negative* (38.6%) in the control group and the *optimistic* (67.9%) in the intervention group. In short, the results suggested that the intervention group showed more optimal transitions—79.3% from negative to positive profiles—compared to the control group, in which 30.8% of the participants shifted from negative to positive profiles.

**Table 3:** Transition probabilities for the latent transition analysis for control and intervention groups

*Transition probabilities to time 2 profiles*					
Control	Negatives	Present/future negatives	Past negatives	Optimists	Positives
Profile 1	0.711	0.237	0.000	0.000	0.052
Profile 2	0.096	0.785	0.000	0.008	0.111
Profile 3	0.092	0.000	0.652	0.256	0.000
Profile 4	0.157	0.140	0.125	0.397	0.181
Profile 5	0.041	0.062	0.000	0.027	0.871

Intervention	Negatives	Present/future negatives	Past negatives	Optimists	Positives
Profile 1	0.847	0.124	0.000	0.000	0.029
Profile 2	0.103	0.676	0.000	0.032	0.189
Profile 3	0.094	0.000	0.363	0.543	0.000
Profile 4	0.131	0.051	0.057	0.690	0.072
Profile 5	0.075	0.051	0.000	0.104	0.770

Table 4 presents the percentages of those individuals who remained within and transitioned between profiles. The movers are defined as individuals who progress or regress across profiles. In general terms, the intervention group featured 31% more progressors and 24% fewer regressors when compared to the control group. The results also showed that the pure profiles (*positives* and *negatives*) exhibited more within-profile stability over time in the intervention group.

**Table 4:** Transitions of adolescents between profiles from pretest to posttest by movers and stayers in the control and intervention groups (%)

	Negatives	Present/future negatives	Past negatives	Optimists	Positives
*Control*					
Regressor	–	9,6	25,6	42,2	13
Stayer	71,1	78,5	62,5	39,7	87,1
Progressor	28,9	11,9	9,2	18,1	–
*Intervention*					
Regressor	–	10,3	9,4	23,9	23
Stayer	84,7	67,6	36,3	69	77
Progressor	15,4	22,1	54,3	7,2	–

### Outcomes of Profile Membership (Explanatory Similarity)

As a final step, an analysis of the relationship between time attitudes profiles and external outcomes, in this case emotional, social, and psychological well-being, can help clarify the differentiation of the profiles. The results showed that the explanatory similarity of the profiles was supported, as the model in which the relations between profiles and outcomes were constrained to equality across measurement times resulted in lower values on all fit indices compared to the model in which these relations were freely estimated (see Table 2). These results suggested that the relations between profiles and outcomes were similar across measurement times. Table 5 reports the means of the outcomes within each profile for each group. The results indicated that the means of each outcome for the most negative profiles (i.e., *negatives* and *present/future negatives*) were lower in the control group compared to the intervention group. Regarding the most central group (i.e., *past negatives*), the means were similar across groups. The *optimists* showed higher means (all outcomes) in the intervention group than in the control group, while the *positives* showed the opposite pattern. However, the means for this *positive* profile were similar across groups.

**Table 5:** Time invariant associations between profile membership and the outcomes by control and intervention group

	Negatives	Present/future negatives	Past negatives	Optimists	Positives	Significant differences (*p* ≤ 0.05)
	Mean [CI]	Mean [CI]	Mean [CI]	Mean [CI]	Mean [CI]	
*Control*						
Emotional Well-being	−1.253 [−1.482; −1.025]	−0.602 [−0.790; −0.414]	−0.017 [−0.293; 0.327]	−0.007 [−0.109; 0.095]	0.405 [0.337; 0.474]	5 > 4 = 3 > 2 > 1
Social Well-being	−1.196 [−1.394; −0.997]	−0.631 [−0.831; −0.431]	−0.046 [−0.517; 0.426]	0.126 [−0.019; 0.271]	0.532 [0.422; 0.643]	5 > 4 = 3 > 2 > 1
Psychological Well-being	−1.383 [−1.664; −1.102]	−0.711 [−0.897; −0.524]	0.053 [−0.266; 0.372]	0.040 [−0.091; 0.171]	0.503 [0.413; 0.592]	5 > 3 = 4 > 2 > 1
*Intervention*						
Emotional Well-being	−0.564 [−0.822; −0.305]	−0.444 [−0.611; −0.276]	−0.035 [−0.301; 0.231]	0.280 [0.184; 0.377]	0.308 [0.204; 0.411]	5 > 4 > 3 > 2 > 1
Social Well-being	−0.529 [−0.983; −0.074]	−0.488 [−0.739; −0.238]	−0.067 [−0.347; 0.213]	0.427 [0.239; 0.615]	0.349 [0.207; 0.492]	4 > 5 > 3 > 2 > 1
Psychological Well-being	−0.698 [−1.006; −0.390]	−0.635 [−0.850; −0.420]	0.064 [−0.319; 0.448]	0.385 [0.271; 0.500]	0.421 [0.315; 0.528]	5 > 4 > 3 > 2 > 1

*Note:* CI: 95% confidence interval

Concerning the intervention group, the *negative* profile reported the lowest levels for all well-being outcomes, with psychological well-being as the outcome with the lowest mean. In contrast, the *positive* profile reported the highest means for emotional and psychological well-being, and the *optimistic* profile reported the highest means for social well-being. The *past negative* was the profile with intermediate results in all outcomes; it showed negative means (close to 0) for emotional and social well-being, yet the mean for psychological well-being was positive. Finally, the *present/future negatives* resembled the negatives, but the means were marginally higher in the *negative* profile. The analysis of the outcomes of profile membership showed that time attitudes profiles were differently associated with indicators of well-being, and therefore reinforces the distinguishing features of each profile identified in the current study. Overall, and supporting the hypotheses presented, the results indicated that a multicomponent positive psychology intervention can elicit adolescents to transition to (more) positive profiles. Also, adolescents allocated in the *negative*, *present/future negative*, and *optimist*ic profiles are subsequently associated with a better psychological adjustment compared to the control group.

## Discussion

In the pursuit of understanding well-being during adolescence, it is important to consider adolescents’ (positive or negative) feelings towards the past, present, and future. Time attitudes are to be considered a relevant aspect to examine when facilitating a healthy transition to adulthood. In the present study, it was investigated the extent to which a randomized-controlled multicomponent positive psychology intervention contributed to the transitions among profiles and the effects of these transitions on adolescent’s emotional, social, and psychological well-being. A total of five profiles were identified: *negative*, *present/future negative*, *past negative*, *optimistic*, and *positive*. Results indicated that adolescents who participated in the intervention reported more transitions to positive profiles compared to the control participants. The findings generally indicates that there were more progressors in the intervention group and more regressors in the control group. Regarding the (emotional, social, and psychological) well-being outcomes after the multicomponent positive psychology intervention program, the intervention group showed better psychological adjustment compared to the control group, with the exception of those individuals belonging to the *positive* profile. The transitions among profiles and the relations to well-being in the “pure” *negative* and *positive* profiles should be interpreted with caution.

### Impact of a Multicomponent Positive Psychology Intervention on Time Attitudes: How Do Adolescents Transition Among Profiles?

Adolescents’ time attitudes commonly change over time, and while transitions to negative profiles are more frequent during adolescence, the results showed that the multicomponent positive psychology intervention Get to Know Me+ may help adolescents to transition to more positive profiles. The number and type of profiles identified in the present study (*negative*, *present/future negative*, *past negative*, *optimistic*, and *positive*) was consistent with previous work on adolescents’ time attitudes (Konowalczyk et al., 2019; Tejada-Gallardo et al., 2021). The *negative* and *positive* profiles can be considered as the “pure” profiles because they are respectively characterized by generally negative and positive feelings towards the past, present, and future. The *present/future negative* profile is characterized by having the most negative views of the present, negative views of the future similar to those individuals belonging to the negative profile, and positive views of the past. The *past negative* and the *optimistic* profiles are both characterized by negative views of the past and positive views of the future; however, *past negatives* presented the worst negative view of the past among profiles.

According to the transitions from negative to positive profiles, those who participated in the intervention were more likely to transition to positive profiles than those in the control group, confirming the first hypothesis. More specifically, 21.8% of the intervention participants moved to the *positive* profile, compared to 16.3% of control participants. Similarly, more than half of the intervention participants, compared to 26.4% of the control group, transitioned to the *optimistic* profile. These findings highlight the positive impact of the multicomponent positive psychology intervention in terms of helping adolescents to have adaptive emotional and evaluative feelings about the past, present, and future. Helping adolescents to experience such feelings can be of great importance in transitional stages, in which having positive feelings towards the three time frames can influence adolescents’ behaviors.

Prior work in the field found that a *positive* profile was related not only to enhanced well-being (Tejada-Gallardo et al., 2021) but also to less alcohol use (McKay et al., 2019), better physical self-concept (Konowalczyk et al., 2019), and less involvement in risk-taking behaviors (Mello et al., 2018). Therefore, the introduction of school-based multicomponent positive psychology interventions that influence adolescents’ transitions towards more positive profiles is likely to promote better emotional, social, and psychological adjustment to early adulthood. In general, adolescents tend to focus more on negative feelings towards present experiences (what is happening), their past recollections (what has happened), and their future expectations (what is yet to come; McKay et al., 2019). These aspects are closely related to the intrinsic motivation and the behavioral engagement adolescents have towards goals (Froiland et al., 2020). Hence, nudging adolescents to shift from more negative to positive profiles may help them accomplish their goals, especially in the school context (Miller & Nickerson, 2008).

With regard to transitions from positive to negative profiles, the multicomponent positive psychology intervention produced similar results in both groups. More concretely, the same percentage of adolescents shifted to the “pure” *negative* profile, while the *present/future negative* and *past negative* profiles in the intervention group received half the number of transitions when compared to those identified in the control group. This result can be explained with reference to the common variability in adolescents’ transitioning to more negative profiles over time (Konowalczyk et al., 2018). The profiles with the highest transitions in the intervention and control group were, respectively, the *past negative* (63.7% of between-profiles transitions) and the *optimistic* (60.3% of between-profiles transitions). These findings suggest that the intervention group had more transitions from negative to positive profiles compared to the control group and highlight the impact that the intervention had on profiles that are not “pure”. In fact, the *negatives* and *positives* reported the highest within-profile stability over time; thus, shifting from the “pure” *negative* profile to more adaptive profiles seems challenging, which might explain the difficulty in intervening and nudging adolescents towards a positive transition.

### Impact of a Multicomponent Positive Psychology Intervention on Well-Being: Do Changes in Time Attitudes Influence Well-Being?

The second goal of the study was to test how changes in time attitude profiles following a multicomponent positive psychology intervention influenced adolescents’ emotional, social, and psychological well-being. Significant and meaningful relationships with well-being outcomes were reported. All profiles in the intervention group showed higher means on the three well-being domains, with the exception of the *past negative* and *positive* profiles, which partly confirms the second hypothesis of the study. In fact, the *past negatives* did not report significant results in any of the groups. In agreement with previous research (Tejada-Gallardo et al., 2021; Worrell et al., 2019), the *past negatives* were the most adaptive among the negative profiles while *negatives* and *present/future negatives* were commonly associated with more detrimental outcomes. Also, the *past negative* profile has mixed positive and negative feelings, resembling the *optimistic* profile, which is also supported in the results obtained in the control group where means of well-being domains did not differ between these two profiles. Still, further research would be needed to explore in depth the results of the well-being’ means on the *past negative* profile after a positive intervention since the results were not the excepted. Among the positive profiles in the intervention group, the *optimistic* showed higher and significant means on the three well-being domains compared to the control group, suggesting that the intervention had a more pronounced impact on this profile compared to the *positive*.

Regarding the specific well-being domains, the positive profiles showed greater means in the eudaimonic component of well-being (i.e., social and psychological). This finding resembles the results of a meta-analytic review indicating that school-based multicomponent positive psychology interventions had the greatest effects on psychological well-being (Tejada-Gallardo et al., 2021). Overall, the intervention led to positive profiles becoming better psychologically adjusted, which is linked to adolescents having identified life objectives to accomplish (González-Carrasco et al., 2019).

An interesting conclusion that can be drawn based on the findings is that the participation of *positive* individuals in a multicomponent positive psychology intervention might be counterproductive, as it would be difficult to substantially increase their levels of well-being (Tomyn et al., 2015). This fact would explain why intervention adolescents in the *positive* profile reported similar well-being than those who did not participate. These results contribute to the debate as to whether positive interventions are equally useful for everyone and gives rise to the question of whether multicomponent positive psychology interventions may have more detrimental than beneficial impacts in terms of promoting well-being when it comes to certain profiles. This trend has led applied researchers to allocate students with higher well-being to control groups and those with lower well-being to intervention groups (Sarriera et al., 2017). Determining how best to increase the well-being of students who already exhibit high well-being seems to be an outgoing challenge that will require further study.

### Limitations

Although the findings of this research have contributed to the study of adolescent’s time attitudes and well-being through an evaluation of the impact of a multicomponent positive psychology intervention, the present study is not without limitations. First, the results cannot be generalized due to the small sample size and the fact that the participants consisted exclusively of adolescents from two high schools in Catalonia (Spain). Regarding the gathering of data, these were collected from self-report measures, which can bias responses of participants. It is also important to note that follow-up assessments were not performed in order to determine whether the transitions across profiles and the influence on well-being remained stable or changed over time. Further studies on this topic should be conducted to obtain more generalizable findings and evaluate the impact of multicomponent positive psychology interventions on time attitudes in the long term. Concerning the multicomponent positive psychology intervention Get to Know Me+, the number of sessions might have proven inadequate. Given that previous meta-analyses have established that as more sessions the program has the more prolonged effects will be reported (i.e., more than six sessions; Bolier et al., 2013; Tejada-Gallardo et al., 2020), including more sessions in the program may have resulted in more pronounced long-term effects (Lyubomirsky & Layous, 2013). In the case of the present study, the length of the intervention was adapted based on the limited time that high schools could allocate to this study. Finally, for further interventions, it would be important to consider participants’ personal features, such as motivation to participate in a multicomponent positive psychology intervention and the effort they dedicate towards the intervention (Lyubomirsky & Layous, 2013). In summary, there are details of the program that will need to be redefined before its next implementation.

### Implications of the Findings

The present study provides evidence of the effectiveness of a school-based multicomponent positive psychology intervention in terms of facilitating transitions to more positive time attitude profiles among adolescents, a salient phenomenon in adolescence. School-based multicomponent positive psychology interventions target not only the well-being of adolescents but also their positive and negative feelings towards the past, present, and future. School-based multicomponent positive psychology interventions are fall under the positive education approach, which recognizes the importance of not only the academic achievement but also the optimal psychological development of children and adolescents (Seligman et al., 2009). Schools should ensure a healthy transition to early adulthood and simultaneously care for students’ well-being. The positive education approach seems a promising strategy by which to implement intervention programs intended to promote adolescents’ optimal functioning in an attempt to reduce the incidence of mental disorders commonly reported during this transitional stage. Unfortunately, only one school in Australia has implemented permanent positive education practices as a whole-school approach; these practices have been found to yield positive results (“Greelong Grammar School”; O’Connor & Cameron, 2017). Given that schools should extend their priorities beyond simply promoting academic competence (Cohen, 2006), it is imperative that positive practices can be incorporated into school curricula, as they could serve to promote positive psychological development on the part of adolescents (Seligman et al., 2009), ensure a healthy transition into adulthood (O’Connor et al., 2017), and diminish the onset of psychological disorders associated with this life stage (Suldo et al., 2014).

When introducing a school-based multicomponent positive psychology intervention that takes time attitudes into account, it is important to be aware of adolescents clustered in profiles with an average positive present and future (i.e., *optimists*), as the results suggest that adolescents in this profile have more positive and adaptive feelings towards these time frames and would therefore be more likely to benefit from an intervention. By contrast, it seems to be more difficult to promote the well-being of adolescents who report extremely negative feelings towards the past (i.e., *past negatives*) through a multicomponent positive psychology intervention. Also, future research should focus more specifically on interventions intended to influence transitions of individuals clustered in the “pure” *negative* profile into more positive profiles and thus support their optimal psychological functioning.

## Conclusion

Having positive feeling towards time has been related to increased levels of well-being in adolescent samples, however, there is a lack of evidence supporting the impact that school-based multicomponent positive psychology interventions have on adolescents’ transitions among time attitude profiles and, more specifically, the influence of these changes on the participants’ emotional, social, and psychological well-being. Hence, the present study investigated the previous assumptions through the implementation of the Get to Know Me+ intervention program in the school setting and the use of a person-centered approach (i.e., latent profile and latent transition analysis) in order to examine the data. The multicomponent positive psychology intervention prompted more transitions to positive profiles in the intervention group compared to the control group. *Past negatives* and *present/future negatives* were the profiles that were more likely to transition to positive profiles in the intervention group. The prevalence of adolescents who remained in negative profiles was higher in the control group. The current study also contributes to a growing body of research examining how multicomponent positive psychology interventions influence well-being with regard to adolescents’ time attitude profiles. The multicomponent positive psychology intervention discussed in this study may enhance the well-being of those in the “pure” *negative*, *present/future negative* and *optimistic* profiles. In summary, the present study provides new evidence indicating that adolescents may benefit from a school-based multicomponent positive psychology intervention and also contributes to extending research on the role of time attitude in these interventions.

### Supplementary information

Supplementary Material 1: Data Analytic Plan

Supplementary Material 2: Results

## Appendix: The Get to Know Me+ Program

The Get to Know Me+ is an intervention program consisting of six weekly sessions that target different components of well-being (i.e., well-being, character strengths, positive emotions, gratitude, optimism, and goal setting; see Table S1 for a detailed plan). The primary goals of the program are to enhance the well-being of adolescents and to promote optimal psychological functioning by means of positive education (Waters 2011) and the development of positive feelings towards time (past, present, and future). Each session took approximately an hour and was divided into four parts: (1) an activity intended to allow students to enter a flow state (10 min), (2) a central activity targeting the component of well-being (30 min), (3) a group-class discussion about the activity (15 min), and (4) a closing part—*what is the take-home message*? (5 min). To control for the effects of time and participation, the control group attended a different class while the program was implemented in the intervention group. The psychologists involved in the intervention individually provided the students from the control group the results of the Values in Action (VIA) strengths questionnaire, with the students subsequently sharing their opinions on the results (Table 6).

**Table 6:** Summary of intervention contents

Modules and sessions	Session goals	Procedure and activities
1. Well-being	○ Establish supportive group environment ○ Introduce students to the broad aspects of well-being (Lyubomirsky et al. 2005) and the aspects that determine it	Flow activity: Mind map activity: “Which memories do you relate with your favorite fruit”? Central activity: Mind map activity: “What does well-being mean to you”? Group discussion: “What does well-being mean? Why is it important?” Closing: Suggesting ways of increasing well-being through purposeful thoughts
*Focus on the positive emotions of the present*		
2. Character strengths	○ Define character strengths and virtues (Park et al. 2004) ○ Explore students’ character strengths through the VIA questionnaire and apply them to different situations	Flow activity: Identify the character strengths (using a card game) of their best friend/important person Central activity 1: Identify their own character strengths Central activity 2: Identify and share two character strengths of their group peers using a card game Central activity 3: Select and apply their top two character strengths in three different contexts (family, friends, school) Group discussion: Discuss their previous selections with the class group Closing: Discuss how character strengths are related to well-being and encourage students to use their greatest strengths
3. Dealing with emotions	○ Introduce the components of emotional intelligence (attention, clarity, and regulation) ○ Challenge negative emotions and thoughts through cognitive restructuring and describing past negative experiences (Fava, 1999)	Flow activity: Identify and define each component of emotional intelligence Central activity: Emotional action process. Identify a conflict situation; recognize the emotions, thoughts, and behavior that the situation evoked; finally, suggest and plan more adaptive responses to future similar situations. Group discussion: Share the central activity with the class group Closing: Discuss the importance of acknowledging their freedom to change and adapt their responses to distressful situations and highlight the contribution of positive emotions to well-being
*Turn back to the positive emotions of the past*		
4. Gratitude	○ Introduce gratitude and its contribution to well-being through prosocial behavior (Froh et al., 2009) ○ Connect with and appreciate positive emotions ○ Learn to integrate actions and expressions of gratitude in their daily lives	Flow activity: *The Desert Island*: “What would you bring with you to a desert island?” Importance of reminding themselves of the most important things in their life Central activity: Gratitude letter Group discussion: Voluntarily share the gratitude letter with the class group Closing: Remind why gratitude can be important for improving well-being. Challenge students to make a gratitude visit during the week
*Move forward to the positive emotions of the future*		
5. Optimistic thinking	○ Introduce optimism and optimistic thinking ○ Learn methods to achieve and/or increase an optimistic explanatory style (Sheldon & Lyubomirsky, 2006)	Flow activity: Identify and share an important memory, person, and wish Central activity: My best possible self Group discussion: Voluntarily share the central activity and discuss the importance of optimistic thinking with the class group Closing: Remind students that hope can help them focus on positive goals for their futures and prevent feelings of helplessness through the belief that there are ways to meet those goals
6. Goal setting	○ Compile the activities and exercises learned through the program ○ Frame life in terms of goal establishment and plans to achieve those goals	Flow activity: Go through and revise the previous program activities and exercises via a snowball effect Central activity: Personal action plan: Establish the steps needed to reach their best selves Group discussion: Share the action plan with the class group Closing: Remind students of how all the activities and exercises covered throughout the program helped increase their well-being. Encourage them to keep on practicing and completing all their work.

## Footnotes

1 Post hoc power analysis was calculated using G*Power (v. 3.1.9.7; Faul et al., 2009) to detect small (i.e., 0.20) effect size with the level of alpha = 0.05 and *n* = 220 (the sample size at pretest). The results indicated a power of 0.92. Likewise, the power was 0.91 for the sample size at posttest (*n*= 213), using the same effect size and alpha parameters.
