# Supplementary material for: Impact of a School-Based Multicomponent Positive Psychology Intervention on Adolescents’ Time Attitudes: A Latent Transition Analysis
Source: J Youth Adolesc. 2021 Dec 31;51(5):1002–16. doi: 10.1007/s10964-021-01562-5 (PMC8993706; doi:10.1007/s10964-021-01562-5)
Supplement: Supplementary file 1 — Supplementary Material 1: Data Analytic Plan [file 10964_2021_1562_MOESM1_ESM.docx]

**Supplementary Material 1: Data Analytic Plan**

***Preliminary Analysis***

Confirmatory factor analysis (CFA) models for time attitudes and the outcome of interest (i.e., well-being) were estimated using the robust maximum likelihood (MLR) estimator. This estimator provides standard errors and tests of fit that permit the parameter estimation from non-normality of continuous observed variables (Finney & DiStefano, 2013). Research indicates that this estimator should be used when the number of response categories for each item is equal to or greater than five (Raykov, 2012).

Before saving the factor scores for our main analyses, we first verified that the measurement models operated in the same manner across time points (Time 1 and Time 2: *N* = 213). These models included six factors for time attitudes (past positive, past negative, present positive, present negative, future positive, and future negative) and three factors for well-being (emotional, social, and psychological). Next, we estimated longitudinal models through sequential tests of measurement invariance (Millsap, 2011). For both constructs (time attitudes and well-being), we assessed (1) configural invariance, (2) weak invariance (loadings), (3) strong invariance (loadings and thresholds), and (4) strict invariance (loadings, thresholds, and uniquenesses).

Given the known oversensitivity of the chi-square test of exact fit (χ2) to sample size and minor model misspecifications (Marsh et al., 2005), we relied on goodness-of-fit indices to describe the fit of the alternative models (Hu & Bentler, 1999): the comparative fit index (CFI), the Tucker-Lewis index (TLI), and the root mean square error of approximation (RMSEA). Values greater than .90 for the CFI and TLI indicate an acceptable fit, although values greater than .95 indicate good model–data fit in general. For the RMSEA, values < .08 indicate reasonable model–data fit; however, values < .06 indicate good model–data fit. In a similar vein, chi-square difference tests also have a known sensitivity to sample size and minor model misspecifications, and research recommends combining this information with changes in CFIs and RMSEAs (Chen, 2007) in tests of measurement invariance. The recommended cut-off scores between two subsequent models when attending to support an invariance hypothesis are ∆CFI ≤ .010 and ∆RMSEA ≤ .015.

***Latent Profile Analysis and Latent Transition Analysis***

The number of profiles retained at each measurement time and for each group is predicted based on multiple sources of information, including a consideration of whether the profiles are meaningful and aligned with theory and statistical adequacy (Marsh et al., 2009; Morin, 2016). Several statistical indices can also be used to support the choice of the retained profiles: (1) the Akaïke information criterion (AIC); (2) the consistent AIC (CAIC); (3) the Bayesian information criterion (BIC); (4) the sample-size adjusted BIC (aBIC); (5) the adjusted Lo-Mendell-Rubin’s likelihood ratio test (aLMR); and (6) the bootstrap likelihood ratio test (BLRT). The AIC, CAIC, BIC, and aBIC are used to compare competing models, and lower values indicate an overall better profile solution. However, these indicators often keep improving with the addition of more profiles. In these cases, information criteria should be graphically presented through “elbow plots” to better observe the improvement related to the addition of profiles (Morin et al., 2011). ﻿The aLMR and BLRT were used to compare the models of the *k* profile and the *k* − 1 profile; when these tests are statistically significant, the model with higher profiles should be retained (Nylund et al., 2007). Entropy is an indicator that highlights the precision of the classification of individuals into latent profiles, with values ranging from 0 (lower accuracy) to 1 (higher accuracy; Tein et al., 2013). Although higher values indicate greater precision in the assignment of individuals to profiles, entropy alone should not be used to determine the optimal number of profiles (Lubke & Muthén, 2007).

**Results**

***Preliminary Analysis***

Table S2 reports the results of the CFA models. These results supported the measurement models of time attitudes and the outcome of interest at each time point. Their complete measurement invariance for the longitudinal models across both time points (i.e., strict invariance) was also accepted, as none of the changes in the goodness-of-fit indices exceeded the recommended cut-off values (∆CFI ≤ .010; ∆TLI ≤ .010; ∆RMSEA ≤ .015). To ensure that the latent profiles estimated at each time point were based on fully comparable measures of time attitudes, the factor scores used in the main analyses were saved from the model of strict measurement invariance. The outcome scores (emotional, social, and psychological well-being) of the most invariant model were also saved. Strict measurement invariance is required to ensure that construct measurement remains equivalent across time points for models based on factor scores (e.g., Millsap, 2011). Table S3 reports the correlations estimated between the factor scores retained from the most invariant measurement model.

***Latent Profile Analysis and Latent Transition Analysis***

Fit indices for the latent profile analysis models at both measurement time points are presented in Table S4 (control group) and Table S5 (experimental group). The AIC and aBIC fit indices continued to decrease with the addition of profiles at each time point in both groups. In contrast, in the control group, the CAIC suggested five profiles and the BIC six profiles (Time 1), while, at Time 2, both fit indices suggested five profiles. In the experimental group, the CAIC and BIC suggested four profiles (Time 1), while, at Time 2, the CAIC suggested four profiles and the BIC five profiles. Finally, the aLMR suggested four profiles (Time 1) and three or five profiles (Time 2) for the control group, while, for the intervention group, the fit indices suggested three profiles (Time 1) and five profiles (Time 2). The BLRT suggested six profiles (Time 1) and seven profiles (Time 2) for the control group and seven profiles (Time 1) and five profiles (Time 2) for the experimental group.

We relied on elbow plots to graphically observe the improvement resulting from the addition of profiles (see Figures S1–S4). These figures showed that the improvement in fit reached a clear plateau at around five profiles. However, the four- and six-profile solutions were also examined, and the results supported the five-profile solution at the statistical and theoretical levels. Compared to the four-profile solution, the five-profile solution resulted in the addition of a new significant profile. By contrast, the addition of an additional profile (six profiles) required the division of an existing profile into two smaller ones. The five-profile solution was then retained at both measurement time points and for each group, providing support for the configural similarity of this latent profile analysis. The entropy retained for the control group was .92 (Time 1) and .93 (Time 2), while that for the intervention group was .96 (Time 1) and .97 (Time 2), indicated that the profiles have a high level of accuracy.

**References**

Chen, F. F. (2007). Sensitivity of goodness of fit indexes to lack of measurement invariance. *Structural Equation Modeling*, *14*(3), 464–504. https://doi.org/10.1080/10705510701301834

Finney, S. J., & DiStefano, C. (2013). Non-normal and categorical data in structural equation modeling. In R. O. M. Hancock (Ed.), *Structural equation modeling: A second course* (pp. 439–492).

Hu, L. T., & Bentler, P. M. (1999). Cutoff criteria for fit indexes in covariance structure analysis: Conventional criteria versus new alternatives. *Structural Equation Modeling*, *6*(1), 1–55. https://doi.org/10.4324/9780203821961

Lubke, G., & Muthén, B. O. (2007). Performance of factor mixture models as a function of model size, covariate effects, and class-specific parameters. Structural Equation Modeling, 14(1), 26–47. https://doi.org/10.1207/s15328007sem1401_2

Marsh, H. W., Hau, K. T., & Grayson, D. (2005). Goodness of fit in structural equation models. In Maydeu-Olivares & J.J. McArdle (Ed.), *Contemporary Psychometrics: A Festschrift for Roderick P.McDonald* (pp. 275–340). https://doi.org/10.4324/9781410612977

Marsh, H. W., Lüdtke, O., Trautwein, U., & Morin, A. J. S. (2009). Classical latent profile analysis of academic self-concept dimensions: Synergy of person- and variable-centered approaches to theoretical models of self-concept. *Structural Equation Modeling*, *16*(2), 191–225. https://doi.org/10.1080/10705510902751010

Millsap, R. E. (2011). Statistical approaches to measurement invariance. In *Statistical Approaches to Measurement Invariance*. Taylor and Francis. https://doi.org/10.4324/9780203821961

Morin, A. J. S. (2016). Person-centered research strategies in commitment research. In John P. Meyer (Ed.), *Handbook of Employee Commitment* (pp. 490–508). Edward Elgar. https://doi.org/10.4337/9781784711740.00050

Morin, A. J. S., Maïano, C., Nagengast, B., Marsh, H. W., Morizot, J., & Janosz, M. (2011). General growth mixture analysis of adolescents’ developmental trajectories of anxiety: The impact of untested invariance assumptions on substantive interpretations. *Structural Equation Modeling*, *18*(4), 613–648. https://doi.org/10.1080/10705511.2011.607714

Nylund, K. L., Asparouhov, T., & Muthén, B. O. (2007). Deciding on the number of classes in latent class analysis and growth mixture modeling: A Monte Carlo simulation study. *Structural Equation Modeling*, *14*(4), 535–569. https://doi.org/10.1080/10705510701575396

Raykov, T. (2012). Scale construction and development using structural equation modeling. In R. H. Hoyle (Ed.), Handbook of structural equation modeling (p. 472–492). The Guilford Press.

Tein, J. Y., Coxe, S., & Cham, H. (2013). Statistical power to detect the correct number of classes in latent profile analysis. *Structural Equation Modeling*, *20*(4), 640–657. https://doi.org/10.1080/10705511.2013.824781
