# Supplementary material for: Impact of a School-Based Multicomponent Positive Psychology Intervention on Adolescents’ Time Attitudes: A Latent Transition Analysis
Source: J Youth Adolesc. 2021 Dec 31;51(5):1002–16. doi: 10.1007/s10964-021-01562-5 (PMC8993706; doi:10.1007/s10964-021-01562-5)
Supplement: Supplementary file 2 — Supplementary Material 2: Results [file 10964_2021_1562_MOESM2_ESM.docx]

**Supplementary Material 2: Results**

**Table S1.**

| Description | *χ*2_(_*_df_*_)_ | CFI | TLI | RMSEA | 90% CI | *∆χ2* (*df*) | ∆CFI | ∆TLI | ∆RMSEA |
| --- | --- | --- | --- | --- | --- | --- | --- | --- | --- |
| **AATI-TA** |  |  |  |  |  |  |  |  |  |
| Time 1 | 387.960_(237)_ | .941 | .931 | .055 | [.045-.064] | — | — | — | — |
| Time 2 | 403.133_(237)_ | .934 | .923 | .057 | [.048-.067] | — | — | — | — |
| Configural invariance | 958.193_(478)_ | .905 | .890 | .069 | [.062-.075] | — | — | — | — |
| Weak invariance | 986.479_(496)_ | .903 | .892 | .068 | [.062-.074] | 31.008_(18)_ | –.002 | .002 | –.001 |
| Strong invariance | 1025.354_(514)_ | .899 | .891 | .068 | [.062-.074] | 37.291_(18)_ | –.004 | –.001 | .000 |
| Strict invariance | 1037.094_(538)_ | .901 | .899 | .066 | [.060-.072] | 95.216_(24)_ | .002 | .008 | –.002 |
| **MHC-SF** |  |  |  |  |  |  |  |  |  |
| Time 1 | 142.278_(74)_ | .920 | .902 | .066 | [.049-.082] | — | — | — | — |
| Time 2 | 169.237_(74)_ | .920 | .902 | .078 | [.062-.093] | — | — | — | — |
| Configural invariance | 312.544_(148)_ | .920 | .902 | .072 | [.061-.083] | — | — | — | — |
| Weak invariance | 321.807_(159)_ | .921 | .909 | .069 | [.058-.080] | 8.594_(11)_ | .001 | .007 | –.003 |
| Strong invariance | 328.371_(170)_ | .923 | .917 | .066 | [.055-.077] | 11.281_(11)_ | .002 | .008 | –.003 |
| Strict invariance | 350.473_(184)_ | .919 | .920 | .065 | [.055-.075] | 18.574_(14)_ | –.004 | .003 | –.001 |

*Goodness-of-Fit Statistics of the Longitudinal Confirmatory Factor Analytic (CFA) Models*

*Note.* AATI-TA = Adolescent and Adult Time Inventory-Time Attitudes; MHC-SF = Mental Health Continuum-Short Form; *χ*2 = chi-square test of exact fit; *df* = degrees of freedom; CFI = comparative fit index; TLI = Tucker-Lewis index; RMSEA = root mean square error of approximation; 90% CI = 90% confidence interval of the RMSEA; *∆χ*2 = chi-square difference test using MLR manual steps.

**Table S2.**

*Latent Correlations from the Fully Invariant Longitudinal Model*

|  | PsP_T1 | PsN_T1 | PrP_T1 | PrN_T1 | FrP_T1 | FrN_T1 | PsP_T2 | PsN_T2 | PrP_T2 | PrN_T2 | FrP_T2 | FrN_T2 | Em_T1 | So_T1 | Psy_T1 | Em_T2 | So_T2 | Psy_T2 |
| --- | --- | --- | --- | --- | --- | --- | --- | --- | --- | --- | --- | --- | --- | --- | --- | --- | --- | --- |
| PsP_T1 | - |  |  |  |  |  |  |  |  |  |  |  |  |  |  |  |  |  |
| PsN_T1 | -.896^**^ | - |  |  |  |  |  |  |  |  |  |  |  |  |  |  |  |  |
| PrP_T1 | .427^**^ | -.307^**^ | - |  |  |  |  |  |  |  |  |  |  |  |  |  |  |  |
| PrN_T1 | -.370^**^ | .464^**^ | -.825^**^ | - |  |  |  |  |  |  |  |  |  |  |  |  |  |  |
| FrP_T1 | .147^*^ | -.011 | .481^**^ | -.345^**^ | - |  |  |  |  |  |  |  |  |  |  |  |  |  |
| FrN_T1 | -2.59^**^ | .308^**^ | -.510^**^ | .629^**^ | -.645^**^ | - |  |  |  |  |  |  |  |  |  |  |  |  |
| PsP_T2 | .729^**^ | -.688^**^ | .283^**^ | -.290^**^ | .047 | -.198^**^ | - |  |  |  |  |  |  |  |  |  |  |  |
| PsN_T2 | -.625^**^ | .699^**^ | -.280^**^ | .393^**^ | .015 | .259^**^ | -.769^**^ | - |  |  |  |  |  |  |  |  |  |  |
| PrP_T2 | .250^**^ | -.199^**^ | .620^**^ | -.561^**^ | .376^**^ | -.427^**^ | .413^**^ | -.241^**^ | - |  |  |  |  |  |  |  |  |  |
| PrN_T2 | -.230^**^ | .285^**^ | -.598^**^ | .685^**^ | -.289^**^ | .521^**^ | -.275^**^ | .499^**^ | -.708^**^ | - |  |  |  |  |  |  |  |  |
| FrP_T2 | -.059 | .131 | .237^**^ | -.202^**^ | .546^**^ | -.437^**^ | .147^*^ | .057 | .561^**^ | -.308^**^ | - |  |  |  |  |  |  |  |
| FrN_T2 | -.143^*^ | .196^**^ | -.397^**^ | .505^**^ | -.345^**^ | .595^**^ | -.215^**^ | .455^**^ | -.418^**^ | .758^**^ | -.481^**^ | - |  |  |  |  |  |  |
| Em_T1 | .351^**^ | -.264^**^ | .729^**^ | -.627^**^ | .493^**^ | -.504^**^ | .224^**^ | -.203^**^ | .584^**^ | -.519^**^ | .317^**^ | -.379^**^ | - |  |  |  |  |  |
| So_T1 | .373^**^ | -.297^**^ | .575^**^ | -.511^**^ | .509^**^ | -.456^**^ | .212^**^ | -.167^*^ | .454^**^ | -.348^**^ | .294^**^ | -.245^**^ | .853^**^ | - |  |  |  |  |
| Psy_T1 | .336^**^ | -.275^**^ | .709^**^ | -.635^**^ | .504^**^ | -.567^**^ | .208^**^ | -.179^**^ | .570^**^ | -.496^**^ | .357^**^ | -.390^**^ | .921^**^ | .850^**^ | - |  |  |  |
| Em_T2 | .317^**^ | -.245^**^ | .547^**^ | -.481^**^ | .384^**^ | -.424^**^ | .399^**^ | -.258^**^ | .703^**^ | -.501^**^ | .455^**^ | -.391^**^ | .670^**^ | .569^**^ | .633^**^ | - |  |  |
| So_T2 | .310^**^ | -.217^**^ | .463^**^ | -.406^**^ | .470^**^ | -.455^**^ | .338^**^ | -.177^**^ | .589^**^ | -.379^**^ | .493^**^ | -.340^**^ | .613^**^ | .645^**^ | .599^**^ | .868^**^ | - |  |
| Psy_T2 | .267^**^ | -.201^**^ | .545^**^ | -.489^**^ | .429^**^ | -.480^**^ | .358^**^ | -.232^**^ | .672^**^ | -.510^**^ | .518^**^ | -.453^**^ | .658^**^ | .587^**^ | .668^**^ | .927^**^ | .905^**^ | - |

*Note:* ^**^*p* < .01, ^*^*p* < .05; PsP = Past Positive; PsN = Past Negative; PrP = Present Positive; PrN = Present Negative; FrP = Future Positive; FrN = Future Negative; Em = Emotional Well-being; So = Social Well-being; Psy = Psychological Well-being; T1 = First point of measurement; T2 = Second point of measurement

**Table S3**.

*Results from the Latent Profile Analysis Models Estimated Separately at Each Time Wave for the Control Group*

|  | LL | #*fp* | Scaling | AIC | CAIC | BIC | aBIC | aLMR *p* | BLRT *p* | Entropy |
| --- | --- | --- | --- | --- | --- | --- | --- | --- | --- | --- |
| **Time 1** |  |  |  |  |  |  |  |  |  |  |
| 1 profile | -842.273 | 12 | 0.932 | 1708.545 | 1755.320 | 1743.320 | 1705.361 | Na | Na | Na |
| 2 profiles | -614.660 | 25 | 1.063 | 1279.320 | 1376.766 | 1351.766 | 1272.685 | <.001 | <.001 | .934 |
| 3 profiles | -550.292 | 38 | 0.902 | 1176.584 | 1324.702 | 1286.702 | 1166.499 | <.001 | <.001 | .941 |
| 4 profiles | -500.004 | 51 | 0.917 | 1102.009 | 1300.799 | 1249.799 | 1088.473 | .042 | <.001 | .940 |
| 5 profiles | -470.052 | 64 | 0.993 | 1068.104 | 1317.566 | 1253.566 | 1051.119 | .352 | <.001 | .917 |
| 6 profiles | -432.532 | 77 | 0.968 | 1019.065 | 1319.199 | 1242.199 | 998.629 | .142 | <.001 | .943 |
| 7 profiles | -414.567 | 90 | 0.885 | 1009.134 | 1359.940 | 1269.940 | 985.248 | .428 | .666 | .961 |
| **Time 2** |  |  |  |  |  |  |  |  |  |  |
| 1 profile | -893.987 | 12 | 1.008 | 1811.974 | 1858.748 | 1846.748 | 1808.789 | Na | Na | Na |
| 2 profiles | -679.122 | 25 | 1.071 | 1408.245 | 1505.691 | 1480.691 | 1401.610 | <.001 | <.001 | .955 |
| 3 profiles | -576.449 | 38 | 0.935 | 1228.898 | 1377.016 | 1339.016 | 1218.813 | <.001 | <.001 | .976 |
| 4 profiles | -532.765 | 51 | 1.086 | 1167.530 | 1366.320 | 1315.320 | 1153.995 | .429 | <.001 | .911 |
| 5 profiles | -489.839 | 64 | 1.092 | 1107.679 | 1357.141 | 1293.141 | 1090.693 | <.001 | <.001 | .926 |
| 6 profiles | -460.671 | 77 | 0.989 | 1075.342 | 1375.906 | 1298.475 | 1054.906 | .559 | <.001 | .940 |
| 7 profiles | -437.620 | 90 | 0.922 | 1055.239 | 1406.045 | 1316.045 | 1031.354 | .009 | <.001 | .954 |

*Note:* Na = not applicable; LL = Model LogLikelihood; #fp = Number of free parameters; Scaling = scaling factor; AIC = Akaïke Information Criteria; CAIC = Consistent AIC; BIC = Bayesian Information Criteria; aBIC = Sample-Size adjusted BIC; aLMR = adjusted Lo-Mendell-Rubin likelihood ratio test; BLRT = Bootstrap Likelihood Ratio Test.

**Table S4.**

*Results from the Latent Profile Analysis Models Estimated Separately at Each Time Wave for the Experimental Group*

|  | LL | #*fp* | Scaling | AIC | CAIC | BIC | aBIC | aLMR *p* | BLRT *p* | Entropy |
| --- | --- | --- | --- | --- | --- | --- | --- | --- | --- | --- |
| Time 1 |  |  |  |  |  |  |  |  |  |  |
| 1 profile | -494.246 | 12 | 0.919 | 1012.491 | 1052.925 | 1040.925 | 1003.088 | Na | Na | Na |
| 2 profiles | -390.691 | 25 | 1.073 | 831.381 | 915.618 | 890.618 | 811.791 | .001 | <.001 | .942 |
| 3 profiles | -355.939 | 38 | 0.927 | 787.877 | 915.916 | 877.916 | 758.100 | .061 | <.001 | .975 |
| 4 profiles | -324.563 | 51 | 0.882 | 751.126 | 922.968 | 871.968 | 711.162 | .105 | <.001 | .982 |
| 5 profiles | -299.749 | 64 | 0.983 | 727.498 | 943.242 | 879.142 | 677.347 | .607 | <.001 | .955 |
| 6 profiles | -273.110 | 77 | 0.948 | 700.220 | 959.667 | 882.667 | 639.882 | .310 | <.001 | .963 |
| 7 profiles | -257.740 | 90 | 0.839 | 695.480 | 998.730 | 908.730 | 624.955 | .297 | <.001 | .956 |
| Time 2 |  |  |  |  |  |  |  |  |  |  |
| 1 profile | -506.247 | 12 | 0.845 | 1036.493 | 1076.927 | 1064.927 | 1027.090 | Na | Na | Na |
| 2 profiles | -408.875 | 25 | 0.858 | 867.750 | 951.986 | 926.986 | 848.160 | <.001 | <.001 | .985 |
| 3 profiles | -364.077 | 38 | 0.943 | 804.154 | 932.193 | 894.193 | 774.377 | .077 | <.001 | .947 |
| 4 profiles | -327.968 | 51 | 0.844 | 757.935 | 929.777 | 878.777 | 717.972 | .039 | <.001 | .967 |
| 5 profiles | -299.773 | 64 | 0.906 | 727.546 | 943.190 | 879.190 | 677.395 | .125 | <.001 | .967 |
| 6 profiles | -280.954 | 77 | 0.908 | 715.907 | 975.355 | 898.355 | 655.570 | .400 | .034 | .969 |
| 7 profiles | 258.527 | 90 | 0.831 | 697.053 | 1000.303 | 910.304 | 626.529 | .873 | .076 | .980 |

*Note:* Na = not applicable; LL = Model LogLikelihood; #fp = Number of free parameters; Scaling = scaling factor; AIC = Akaïke Information Criteria; CAIC = Consistent AIC; BIC = Bayesian Information Criteria; aBIC = Sample-Size adjusted BIC; aLMR = adjusted Lo-Mendell-Rubin likelihood ratio test; BLRT = Bootstrap Likelihood Ratio Test.

**Table S5.**

*Detailed Results from the Final Latent Transition Solution (Dispersion Similarity)*

|  | Profile 1 (11,26%) | | Profile 2 (17,37%) | | Profile 3 (9,85%) | | Profile 4 (15,49%) | | Profile 5 (46%) | |
| --- | --- | --- | --- | --- | --- | --- | --- | --- | --- | --- |
|  | Mean | CI | Mean | CI | Mean | CI | Mean | CI | Mean | CI |
| PsP | -.699 | [-.870; -.527] | .207 | [.077; .463] | -.1520 | [-2.036; -1.004] | -.550 | [-.778; -.322] | .606 | [.546; .667] |
| PsN | 1.195 | [.956; 1.433] | -.426 | [-.583; -.270] | 1.960 | [1.504; 2.417] | .587 | [.107; 1.068] | -.706 | [-.749; -.664] |
| PrP | -.719 | [-.883; -.555] | -.762 | [-.979; -.546] | .071 | [-.174; .317] | -.002 | [-.177; .174] | .563 | [.478; .648] |
| PrN | .924 | [.811; 1.038] | .507 | [.377; .637] | .107 | [-.127; .342] | .046 | [-.135; .228] | -.403 | [-.458; -.349] |
| FrP | -.363 | [-.486; -.239] | -.406 | [-.517; -.294] | .396 | [.264; .529] | .118 | [-.120; .356] | .168 | [.102; .235] |
| FrN | 1.118 | [.941; 1.294] | .414 | [.283; .545] | -.137 | [-.300; .026] | -.030 | [-.183; .124] | -.314 | [-.371; -.258] |

*Note:* PsP = Past Positive; PsN = Past Negative; PrP = Present Positive; PrN = Present Negative; FrP = Future Positive; FrN = Future Negative; Profile 1 = Negatives; Profile 2 = Present/Future Negatives; Profile 3 = Past Negatives; Profile 4 = Optimists; Profile 5 = Positives

**Figure S1.**

*Elbow Plot of the Information Criteria for the Latent Profile Analyses at Time 1 (Control group)*


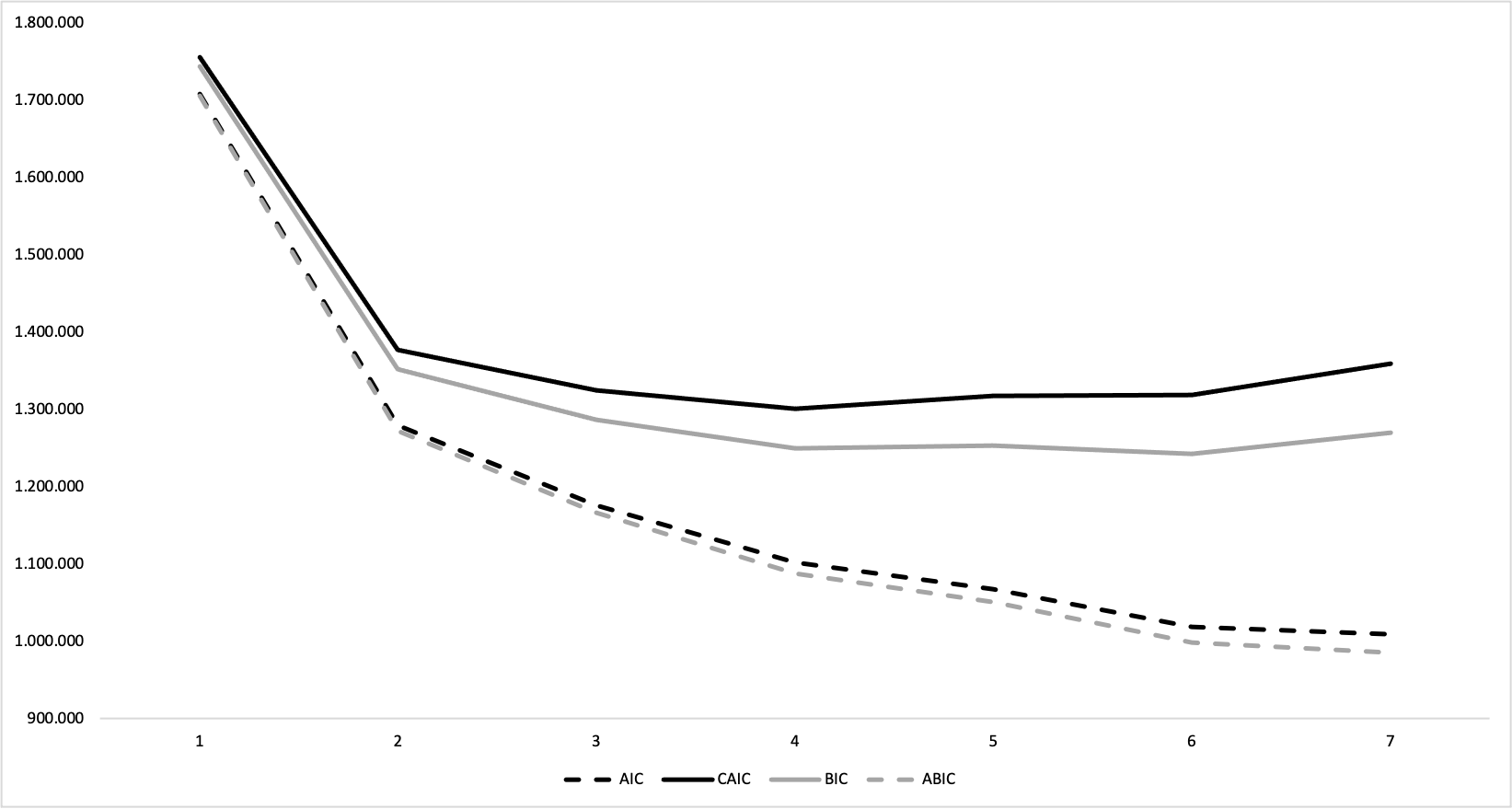


**Figure S2.**

*Elbow Plot of the Information Criteria for the Latent Profile Analyses at Time 1 (Experimental group)*


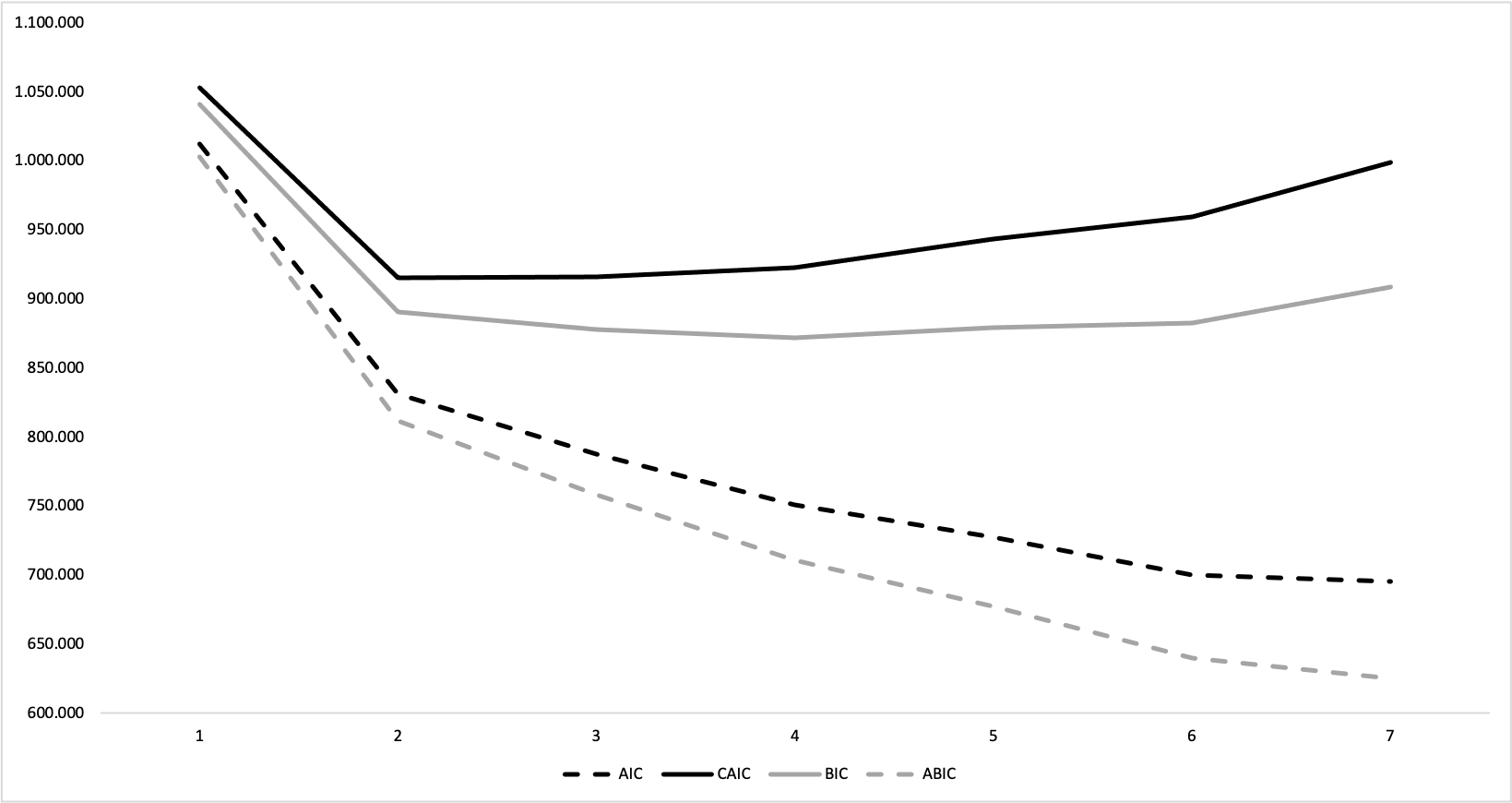


**Figure S3.**

*Elbow Plot of the Information Criteria for the Latent Profile Analyses at Time 2 (Control Group)*


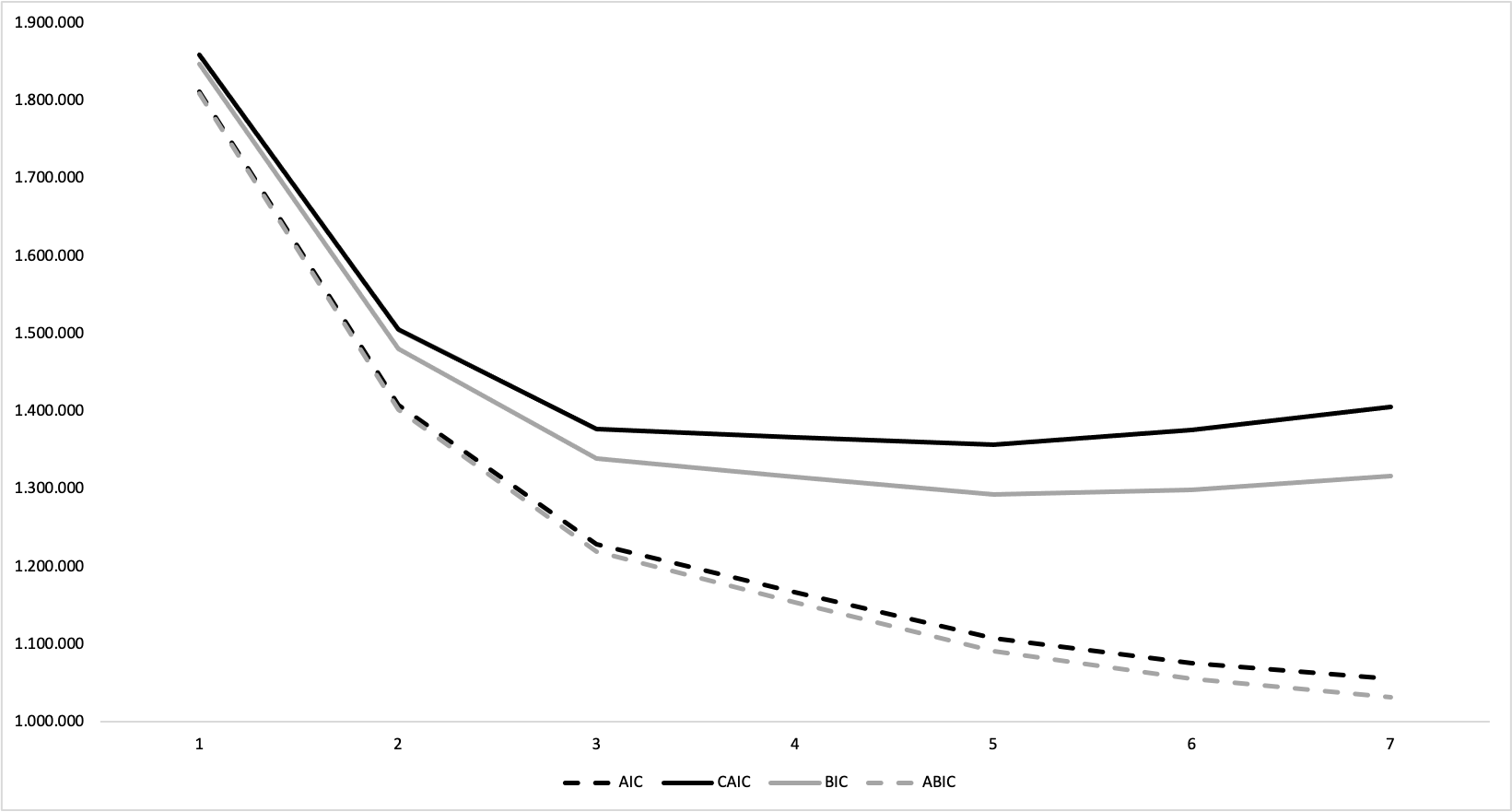


**Figure S4.**

*Elbow Plot of the Information Criteria for the Latent Profile Analyses at Time 2 (Experimental group)*

**
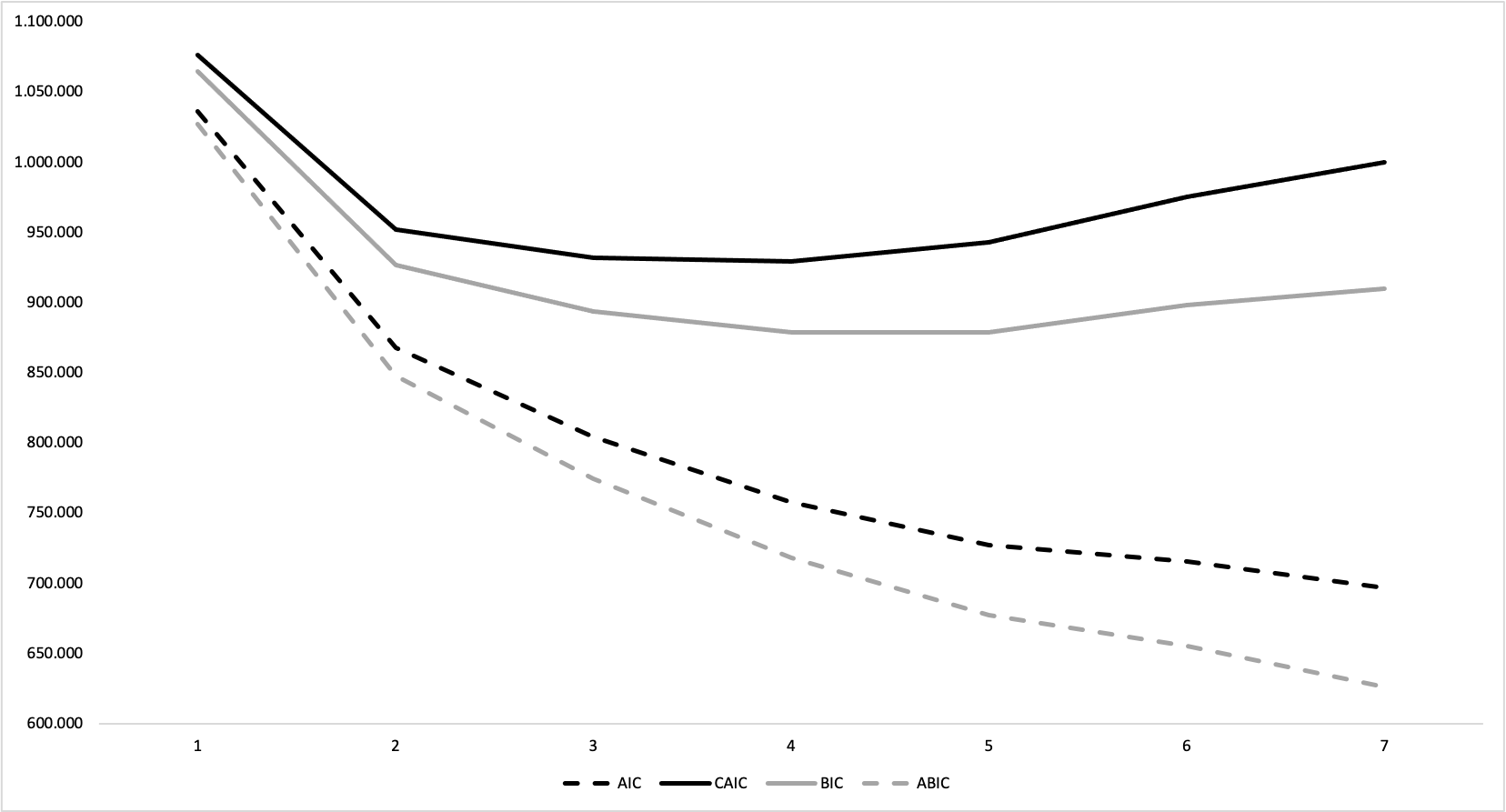
**
